# Elevated osteopontin and IFNy serum levels and increased neutrophil-to-lymphocyte ratio are associated with the severity of symptoms in schizophrenia

**DOI:** 10.1192/j.eurpsy.2022.302

**Published:** 2022-09-01

**Authors:** M. Kovács, T. Tényi, R. Kugyelka, L. Prenek, R. Herold, P. Balogh, D. Simon

**Affiliations:** 1 Medical Faculty, University of Pécs, Hungary, Department Of Psychiatry And Psychotherapy, Pécs, Hungary; 2 Medical Faculty, University of Pécs, Hungary, Department Of Immunology And Biotechnology, Pécs, Hungary

**Keywords:** osteopontin, Cytokines, inflammation, schizophrénia

## Abstract

**Introduction:**

Inflammation and immune dysregulation could contribute to the pathogenesis of schizophrenia. Osteopontin (OPN) is a key cytokine-like molecule in cellular immune response and it can directly modulate the cytokine expression and survival of microglia. Furthermore, its mRNA expression is elevated in first episode psychosis. Imbalance of T-helper subtypes could also represent a vulnerability factor for schizophrenia.

**Objectives:**

The aim of this study was to evaluate the relevance of T-helper subtype associated cytokines, OPN and NLR in the assessment of the severity of schizophrenia.

**Methods:**

22 patients with schizophrenia were assessed for the intensity of their symptoms by PANSS and CGI scores. Serum OPN, IFNy, IL-10 and IL-8 concentrations were measured by ELISA kits and NLR was calculated from blood count. Statistical evaluation was performed using Mann-Whitney U test, Student’s t test and Spearman correlation.

**Results:**

We found significant correlation between the level of OPN and PANSS-total, PANSS-general scores. IFNy level and NLR showed significant correlation with PANSS-total, PANSS-positive, PANSS-general and CGI score. Antipsychotic therapy only had significant effects on NLR and OPN levels, both of which were significantly reduced after long-term antipsychotic treatment.

**Conclusions:**

Our results indicate that elevated OPN and IFNy concentrations, and increased NLR are associated with severe symptoms in schizophrenia and suggest the importance of Th1 subtype in patients with high PANSS-positive and PANSS-general score. Antipsychotic treatment had significant effects on the level of OPN and NLR, but not on the level of IFNy. Overall our results strengthen the inflammation hypothesis of schizophrenia.

**Disclosure:**

No significant relationships.

